# Supplementary material for: The Improvement of Adaptive Immune Responses towards COVID-19 Following Diphtheria–Tetanus–Pertussis and SARS-CoV-2 Vaccinations in Indonesian Children: Exploring the Roles of Heterologous Immunity
Source: Vaccines (Basel). 2024 Sep 9;12(9):1032. doi: 10.3390/vaccines12091032 (PMC11435621; doi:10.3390/vaccines12091032)
Supplement: Supplementary file 1 [file vaccines-12-01032-s001.zip › vaccines-3122953-supplementary.pdf]

Supplementary Table S1. Characteristics of study subjects as stratified in four groups.

**Parameter**	**Group A (n = 39)**	**Group B (n = 38)**	**Group C (n = 38)**	**Group D (n = 39)**	**p-value**
History of acute respiratory infection in last 6 months [n (%)]					
< 3 times (n = 106)	22 (20.8%)	26 (24.5%)	28 (26.4%)	30 (28.3%)	0.220
≥ 3 times (n= 48)	17 (35.4%)	12 (25.0%)	10 (20.8%)	9 (18.9%)	
History of COVID-19 disease in other family members [n (%)]					
No	15 (20.5%)	16 (21.9%)	22 (30.1%)	20 (27.4%)	0.309
Yes	24 (29.6%)	22 (27.2%)	16 (19.8%)	19 (23.5%)	

Group A = COVID-19 yes / DTP yes. Group B = COVID-19 yes / DTP no. Group C = COVID-19 no / DTP yes. Group D = COVID-19 no / DTP no. Kruskal-Wallis test was performed. p-value <0.05 was considered as statistical significant.

Supplementary Table S2. Titers of anti-SARS-CoV-2 S-RBD antibodies among the study participants.

Group	n	Anti-SARS-CoV-2 S-RBD (U/mL) median (min–max)	p-value
A [COVID-19 yes / DTP yes]	39	1,196 (16–15,561)	0.089
B [COVID-19 yes / DTP no]	38	771.2 (0.3–14,589)	
C [COVID-19 no / DTP yes]	38	1,162.5 (0.3–22,269)	
D [COVID-19 no / DTP no]	39	527.9 (0.3–9,060)	

Kruskal-Wallis test was performed with p-value <0.05 was considered as statistically significant.

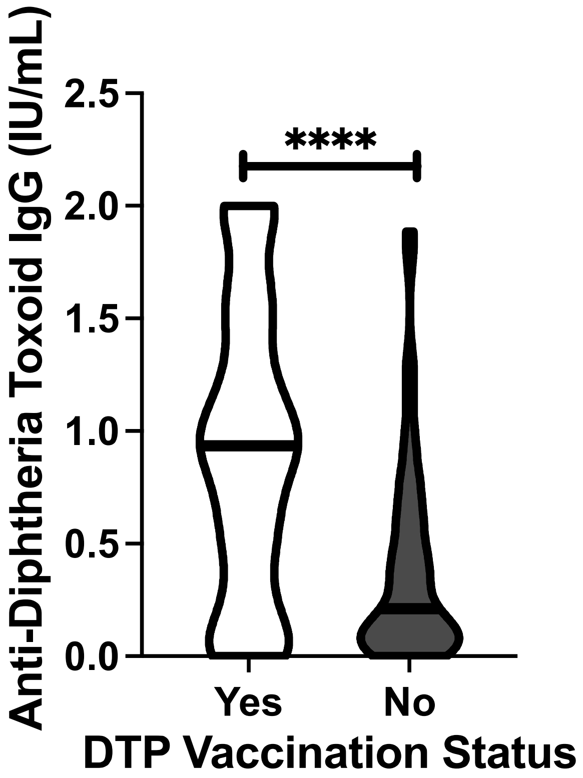

Supplementary Figure S1. Titers of anti-diphtheria toxoid immunoglobulin G of all participants. Study participants were classified into two groups based on their DTP vaccination status, i.e., Yes (had received 3 doses of DTP primary vaccine with 1 dose of DT booster vaccine at 5 years old, irrespective whether receiving an additional dose of DTP vaccine before 2 years old) and No (had only received 3 doses of DTP primary vaccine). Solid horizontal line within each violin plot refers to the median value. Mann-Whitney test was performed to determine any statistical difference between two groups, in which **** marked $p<0.0001$.

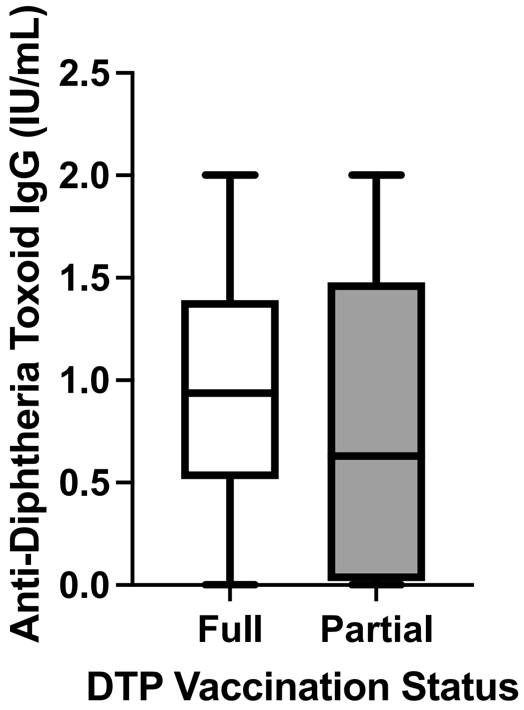

Supplementary Figure S2. Titers of anti-diphtheria toxoid immunoglobulin G of study subjects who had received 3 doses of DTP vaccine and 1 dose of DT booster. Subjects who had received 3 doses of DTP vaccine and 1 dose of DT booster (n=77) were classified into two groups, i.e., *Full* (had also received 1 additional dose of DTP vaccine before 2 years old; n=63) and *Partial* (had not received 1 additional dose of DTP vaccine before 2 years old; n=14). Solid horizontal line within each box-and-whiskers refers to the median value. The whiskers refer to respective minimum and maximum values. Mann-Whitney test was performed with $p<0.05$ was considered as statistically significant.
